# Protective ventilation with high versus low positive end-expiratory pressure during one-lung ventilation for thoracic surgery (PROTHOR): study protocol for a randomized controlled trial

**DOI:** 10.1186/s13063-019-3208-8

**Published:** 2019-04-11

**Authors:** T. Kiss, J. Wittenstein, C. Becker, K. Birr, G. Cinnella, E. Cohen, M. R. El Tahan, L. F. Falcão, C. Gregoretti, M. Granell, T. Hachenberg, M. W. Hollmann, R. Jankovic, W. Karzai, J. Krassler, T. Loop, M. J. Licker, N. Marczin, G. H. Mills, M. T. Murrell, V. Neskovic, Z. Nisnevitch-Savarese, P. Pelosi, R. Rossaint, M. J. Schultz, A. Serpa Neto, P. Severgnini, L. Szegedi, T. Vegh, G. Voyagis, J. Zhong, M. Gama de Abreu, M. Senturk, for the PROTHOR investigators, Vojislava Neskovic, Nevena Radovic, Goran Rondovic, Dusica Stamenkovic, Rade Vukovic, Snjezana Zeba, Rolf Rossaint, Mark Coburn, Ana Kowark, Sebastian Ziemann, Julia van Waesberghe, Wolfgang Bauer, Lotte Terwindt, Kostas Kostopanagiotou, Andreas Kostroglou, Katerina Kyttari, Tatiana Sidiropoulou, María-José Jiménez Andújar, Manuel López-Baamonde, Ricard Navarro Ripoll, Lorena Rivera Vallejo, Matthew Henry, Anita Jegarl, Matthew Murrell, Patrick O’Hara, Michele Steinkamp, Jens Kraßler, Susanne Schäfer, Charlotte Becker, Katja Birr, Thomas Bluth, Marcelo Gama de Abreu, Sara Hattenhauer, Thomas Kiss, Martin Scharffenberg, Robert Teichmann, Jakob Wittenstein, Vitali Costanza, Spadaro Savino, Carlo Alberto Volta, Riccardo Ragazzi, Karim Mariano, Lucia Mirabella, Giuseppina Mollica, Luigi Montrano, Torsten Loop, Axel Semmelmann, Steffen Wirth, Changhong Miao, Jing Zhong, Hu Lv, Hui Wang, Xue Zhang, Yue Zhang, Paolo Pelosi, Laura Corsi, Nicolò Partroniti, Maura Mandelli, Giulia Bonatti, Francesca Simonassi, Angelo Gratarola, Juan José Rodriguez Ruiz, Tania Socorro, Maria Christofaki, Vasileia Nyktari, Alexandra Papaioannou, Nüzhet Mert Şentürk, Emre Bingul, Mukadder Orhan Sungur, Zerrin Sungur, Manuel Heidegger, Vera Dossow, Wiebke Jerichow, Tobias Kammerer, Julia Richter, Barbara Schuba, Eike Speck, Anna-Lisa Stierle, Jan Bruthans, Jan Matek, Pavel Michálek, Loes Didden, Jan Hofland, Marieke Kuut, Jo Mourisse, Sonsoles Aragon, Rafael Esturi, Encarna Miñana, Fernando Sanchez, Elaine Sfikas, Athanasios Kapezanos, Konstantinos Papamichail, Levon Toufektzian, Gregorios Voyagis, Manuel Granell Gil, Asunción Vergara Sánchez, José De Andres, Javier Morales Sarabia, Ana Broseta Lleó, Javier Hernández Laforet, Mercedes Murcia Anaya, Denis Pereira Matalobos, Pilar Aguirre Puig, Jasna Špiček Macan, Vjekoslav Karadza, Nevenka Kolaric, Lea Andjelković, Mojca Drnovšek Globokar, Kristina Gorjup, Ana Mavko, Dejan Pirc, Cadar Genoveva, Raluca Istrate, Radu Stoica, Dan Corneci, Narcis Valentin Tanase, Jankovic Radmilo, Vladan Cvetanovic, Vesna Dinic, Tijana Grbesa, Katarina Jovic, Aleksandar Nikolic, Milena Stojanovic, Ines Veselinovic, Anita Vukovic, Frank Wappler, Jerome Michel Defosse, Stefanie Wehmeier, Thomas Ermert, Carola Wempe, Manuel Wenk, Daniela Iolanda Ion, Cristian Ionescu, Thomas Schilling, Tamar Macharadze, Pei-Ching Li, Yi-Ting Chang, Alberto Noto, Placido Calì, Giovanni Desalvo, Raffaele Deluca, Nicola Giofre, the Research Workgroup PROtective VEntilation Network (PROVEnet) of the European Society of Anaesthesiology (ESA)

**Affiliations:** 1 Department of Anesthesiology and Intensive Care Medicine, Pulmonary Engineering Group, University Hospital Carl Gustav Carus, Technische Universität Dresden, Dresden, Germany; 2 Department of Anesthesia and Intensive Care, OO Riuniti Hospital, University of Foggia, Foggia, Italy; 3 Department of Anesthesiology, The Mount Sinai Hospital, New York, USA; 4 Imam Abdulrahman Bin Faisal University, Dammam, Saudi Arabia; 5 Federal University of São Paulo, Sao Paulo, Brazil; 6 UOC Anestesia e Rianimazione A.O.Universitaria “P. Giaccone”, Dipartimento Di.Chir.On.S., Università degli Studi di Palermo, Palermo, Italy; 7 Hospital General Universitario de Valencia, Valencia, Spain; 8 University Hospital Magdeburg, Magdeburg, Germany; 9 Department of Anesthesiology, Amsterdam UMC, location AMC, Amsterdam, The Netherlands; 10 Clinic for Anesthesia and Intensive Therapy, Clinical Center Nis, School of Medicine, University of Nis, Nis, Serbia; 11 Zentralklinik Bad Berka, Bad Berka, Germany; 12 Thoracic Center Coswig, Coswig, Germany; 13 Department of Anesthesiology and Intensive Care Medicine Clinic, Medical Center, University of Freiburg, Faculty of Medicine, University of Freiburg, Freiburg, Germany; 14 University Hospital Geneva, Geneva, Switzerland; 15 Section of Anaesthetics, Pain Medicine and Intensive Care, Department of Surgery and Cancer, Faculty of Medicine, Imperial College London, London, UK; 16 Department of Anaesthesia, Royal Brompton and Harefield NHS Foundation Trust, Harefield Hospital, Harefield, Middlesex, UK; 17 Centre of Anaesthesia and Intensive Care, Semmelweis University, Budapest, Hungary; 18 Department of Anaesthesia and Intensive Care Medicine, Sheffield Teaching Hospitals, Sheffield University, Sheffield, UK; 19 Department of Anesthesiology, Weill Cornell Medicine, New York, USA; 20 Military Medical Academy, Belgrade, Serbia; 21 Penn State Hershey Anesthesiology & Perioperative Medicine, Hershey, USA; 22 Department of Surgical Sciences and Integrated Diagnostics, University of Genoa, Genoa, Italy; 23 IRCCS San Martino Policlinico Hospital, Genoa, Italy; 24 Department of Anaesthesiology, University Hospital Aachen, Aachen, Germany; 25 Department of Intensive Care & Laboratory of Experimental Intensive Care and Anesthesiology (L·E·I·C·A), Academic Medical Center, University of Amsterdam, Amsterdam, The Netherlands; 26 Mahidol-Oxford Tropical Medicine Research Unit (MORU), Mahidol University, Bangkok, Thailand; 27 Department of Critical Care, Hospital Israelita Albert Einstein, São Paulo, Brazil; 28 Dipartimento di Biotecnologie e Scienze della Vita, Università degli Studi dell’Insubria, Varese, Italy; 29 Department of Anesthesiology, Centre Hospitalier Universitaire de Charleroi, Charleroi, Belgium; 30 Department of Anesthesiology and Intensive Care, University of Debrecen, Debrecen, Hungary; 31 Outcomes Research Consortium, Cleveland, USA; 32 Department of Anaesthesia, Postoperative ICU, Pain Relief & Palliative Care Clinic, “Sotiria” Chest Diseases Hospital, Athens, Greece; 33 Department of Anaesthesiology and Critical Care Medicine, University of Patras, Patra, Greece; 34 Department of Anesthesiology, Fudan University Shanghai Cancer Center, Shanghai, China; 35 Department of Oncology, Shanghai Medical College, Fudan University, Shanghai, China; 36 Department of Anaesthesiology and Intensive Care, Istanbul University, Istanbul Medical Faculty, Istanbul, Turkey

**Keywords:** Mechanical ventilation, positive end-expiratory pressure, recruitment maneuver, one-lung ventilation, thoracic surgery, postoperative pulmonary complication

## Abstract

**Background:**

Postoperative pulmonary complications (PPC) may result in longer duration of in-hospital stay and even mortality. Both thoracic surgery and intraoperative mechanical ventilation settings add considerably to the risk of PPC. It is unclear if one-lung ventilation (OLV) for thoracic surgery with a strategy of intraoperative high positive end-expiratory pressure (PEEP) and recruitment maneuvers (RM) reduces PPC, compared to low PEEP without RM.

**Methods:**

PROTHOR is an international, multicenter, randomized, controlled, assessor-blinded, two-arm trial initiated by investigators of the PROtective VEntilation NETwork. In total, 2378 patients will be randomly assigned to one of two different intraoperative mechanical ventilation strategies. Investigators screen patients aged 18 years or older, scheduled for open thoracic or video-assisted thoracoscopic surgery under general anesthesia requiring OLV, with a maximal body mass index of 35 kg/m^2^, and a planned duration of surgery of more than 60 min. Further, the expected duration of OLV shall be longer than two-lung ventilation, and lung separation is planned with a double lumen tube. Patients will be randomly assigned to PEEP of 10 cmH_2_O with lung RM, or PEEP of 5 cmH_2_O without RM. During two-lung ventilation tidal volume is set at 7 mL/kg predicted body weight and, during OLV, it will be decreased to 5 mL/kg. The occurrence of PPC will be recorded as a collapsed composite of single adverse pulmonary events and represents the primary endpoint.

**Discussion:**

PROTHOR is the first randomized controlled trial in patients undergoing thoracic surgery with OLV that is adequately powered to compare the effects of intraoperative high PEEP with RM versus low PEEP without RM on PPC. The results of the PROTHOR trial will support anesthesiologists in their decision to set intraoperative PEEP during protective ventilation for OLV in thoracic surgery.

**Trial registration:**

The trial was registered in clinicaltrials.gov (NCT02963025) on 15 November 2016.

**Electronic supplementary material:**

The online version of this article (10.1186/s13063-019-3208-8) contains supplementary material, which is available to authorized users.

## Background

Postoperative pulmonary complications (PPC) increase morbidity, resulting in longer duration of in-hospital stay and even increased mortality [1–3]. Several independent risk factors for the development of PPC have been identified [4], including patients’ health conditions, surgical approaches, and anesthetic management [5]. In addition, thoracic surgery [3] and intraoperative mechanical ventilation settings [2] add considerably to the risk of PPC.

Experimental [6–8] and clinical evidence [9–11] show that mechanical ventilation has the potential to aggravate or even initiate lung injury (so called ventilator-induced lung injury; VILI). Repetitive collapse/reopening of lung units (atelectrauma), overdistension of lung units (volutrauma), and increased airway pressures (barotrauma) are possible mechanisms underlying VILI [12–14]*.* While positive end-expiratory pressure (PEEP) can minimize atelectrauma and low tidal volumes (VT) reduce volutrauma, ventilation at low airway pressures may decrease barotrauma.

A metanalysis showed that use of low VT is associated with favorable outcomes in patients without injured lungs [15]. More recently, another meta-analysis showed a decrease in the incidence of lung injury, pulmonary infection, and atelectasis in patients receiving intraoperative mechanical ventilation with low VT and PEEP [16]*.* In patients undergoing abdominal surgery, an intraoperative ventilation strategy with low VT and PEEP improved postoperative lung function [17] and even outcome [16]*.* In contrast, when low VT is used, the use of high PEEP combined with recruitment maneuvers (RM), as compared to low PEEP without RM, does not add to protection against PPC [18]*.* To our knowledge, the potential of high PEEP and RM during one-lung ventilation (OLV) for thoracic surgery to reduce PPC has not been investigated in adequately powered trials [19, 20]*.* Due to mediastinal displacement, surgical manipulation, and chest immobilization, pressures in the dependent lung [21] and atelectasis formation are higher during thoracic surgery as compared with the other types of surgeries [22]. Thus, OLV might benefit from mechanical ventilation with high PEEP and RM.

In view of these facts, we designed the PROtective ventilation with high versus low PEEP during OLV for THORacic surgery (PROTHOR) trial. We hypothesized that intraoperative mechanical ventilation using high PEEP with periodic RM, as compared to low PEEP without RM, will prevent PPC in patients undergoing thoracic surgery with OLV.

## Methods

### Objectives and design

**Fig. 1:**
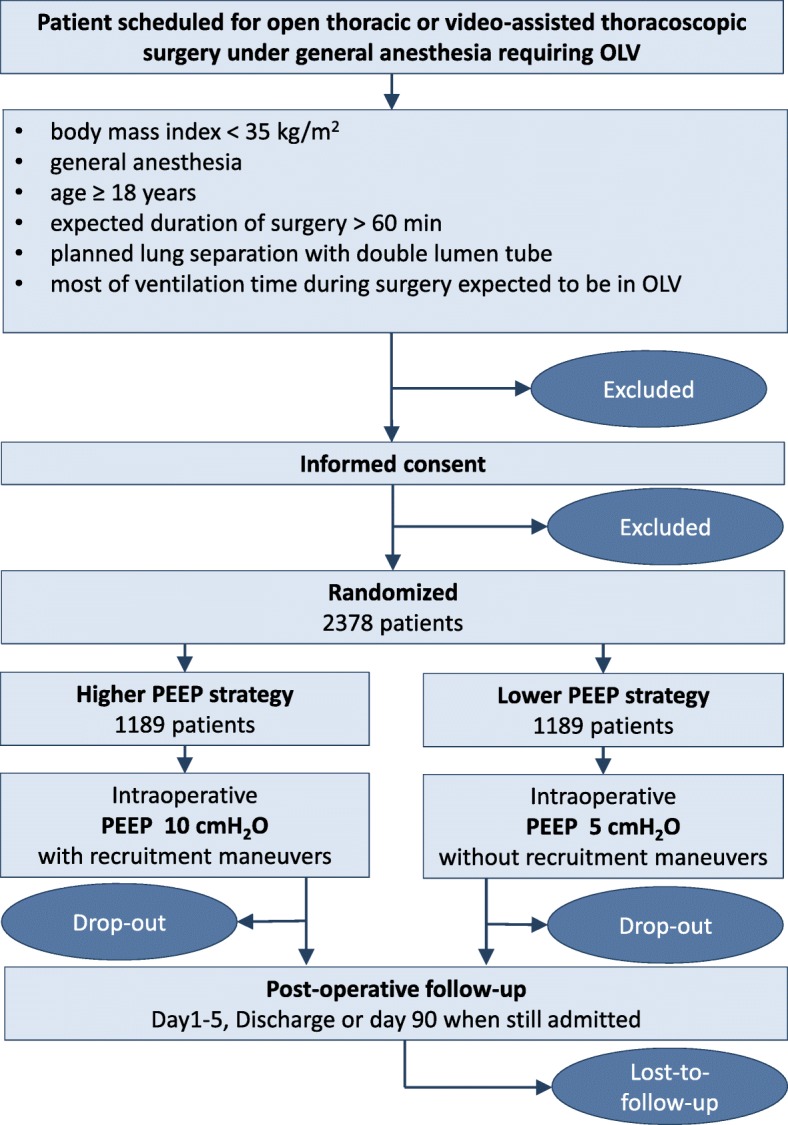
CONSORT Diagram for the PROTHOR trial. *OLV* one-lung ventilation, *PEEP* positive end-expiratory airway pressure

PROTHOR is an international, multicenter, randomized, controlled, assessor-blinded, two-arm trial initiated by investigators of the PROtective VEntilation NETwork (http://provenet.eu). In total, 2378 patients will be randomly assigned to one of two different intraoperative mechanical ventilation strategies (see CONSORT diagram, Fig. 1).

The PROTHOR trial tests the hypothesis that, in patients undergoing thoracic surgery under OLV, high levels of PEEP and RM, as compared with low levels of PEEP without RM, reduce PPC.

#### Study population

Investigators screen patients aged 18 years or above scheduled for open thoracic or video-assisted thoracoscopic surgery under general anesthesia requiring OLV, with a maximal body mass index of 35 kg/m^2^, and a planned duration of surgery of more than 60 min. Further, the expected duration of OLV shall be longer than two-lung ventilation (TLV), and lung separation is planned with a double lumen tube. The number of patients meeting these enrollment criteria will be recorded by means of a screening log file.

Patients are excluded if they have documented chronic obstructive pulmonary disease (COPD) GOLD grades III and IV, lung fibrosis, documented bullae, severe emphysema or pneumothorax; uncontrolled asthma; heart failure New York Heart Association grade 3 and 4 or coronary heart disease Canadian Cardiovascular Society grade 3 and 4; previous lung surgery; at-rest documented mean pulmonary arterial hypertension > 25 mmHg, or systolic pulmonary arterial pressure > 40 mmHg (as estimated by ultrasound); documented or suspected neuromuscular disease (e.g., thymoma, myasthenia, myopathies, muscular dystrophies); are planned for mechanical ventilation after surgery; are planned for bilateral procedures; undergo lung separation with a method other than double lumen tube; are operated in prone position; show persistent hemodynamic instability or intractable shock (as judged by the treating physician); have intracranial injury or tumor; are enrolled in other interventional studies or refuse informed consent; are pregnant (excluded by anamnesis and/or laboratory analysis); have documented preoperative hypercapnia > 45 mmHg (6 kPa, kPa); are planned for esophagectomy, pleural surgery only, sympathectomy surgery only, chest wall surgery only, mediastinal surgery only, and lung transplantation without surgical treatment of the lung tissue. Additionally, patients will be excluded if aspiration, moderate respiratory failure, infiltrates, pulmonary infection, atelectasis, cardiopulmonary edema, pleural effusion, pneumothorax, pulmonary embolism, purulent pleurisy, or lung hemorrhage are diagnosed before surgery.

### Intervention

#### Mechanical ventilation

Mechanical ventilation is applied in volume-controlled mode. Following intubation, PEEP is set according to the randomization group, i.e., 5 cmH_2_O in the low PEEP level group and 10 cmH_2_O in the high PEEP level group. In both groups, the PEEP is maintained unchanged until extubation, unless rescue for hypoxemia mandates adjustments. If auto-PEEP is suspected, the respiratory rate or inspiratory to expiratory time (I:E) ratio may be changed at discretion of the treating physician.

In the high PEEP group, RM are performed at the following occasions:

- after bronchoscopy or disconnection of the ventilated lung from the mechanical ventilator
- at start of OLV
- every 1 hour during OLV
- after re-expansion of the non-dependent lung to resume TLV
- end of surgery in supine position

During TLV, VT is set at 7 mL/kg predicted body weight (PBW). The PBW is calculated according to a predefined formula, as follows: 50 + 0.91 x (height in cm – 152.4) for males and 45.5 + 0.91 x (height in cm – 152.4) for females [23].

During OLV, VT will be decreased to 5 mL/kg PBW, while keeping other settings initially unchanged. If peak pressure > 40 cmH_2_O, or plateau pressure > 30 cmH_2_O, the I:E ratio is first changed to 1:1. Thereafter, VT can be decreased to 4 mL/kg PBW.

Further settings are fraction of inspiratory oxygen (F_I_O_2_) ≥ 0.4, I:E 1:1 to 1:2, and respiratory rate adjusted to normocapnia (partial arterial carbon dioxide pressure (PaCO_2_) between 35 and 45 mmHg).

#### RM and lung expansion maneuvers

**Fig. 2:**
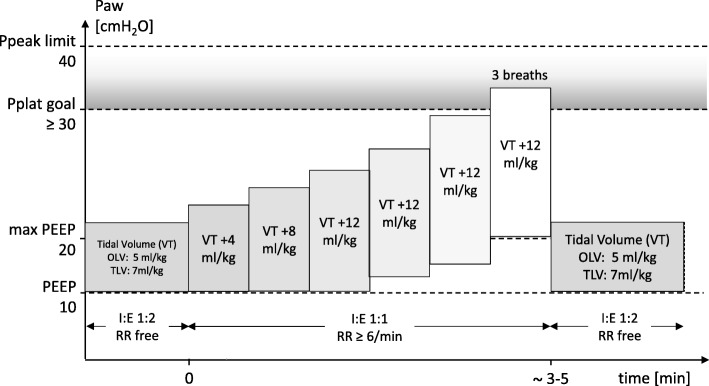
Standardized lung recruitment maneuver in the high PEEP group. *P*_*peak*_ peak airway pressure, *P*_*plat*_ plateau airway pressure, *PEEP* positive end-expiratory airway pressure, *VT* tidal volume normalized for predicted body weight, *RR* respiratory rate, *I:E* ratio between inspiratory and expiratory time

Standardized RM (Fig. 2) are performed with stepwise increase of VT in volume-controlled ventilation (Table 1).

**Table 1:** Recruitment and lung re-expansion maneuver steps

Recruitment maneuver	1. Increase F_I_O_2_ to 1.0 2. Set peak inspiratory pressure limit to 45 cmH_2_O 3. Set respiratory rate to six breaths/min 4. Set I:E ratio to 1:1 5. Increase VT in steps of approximately 2 mL/kg PBW until plateau pressure reaches 30–40 cmH_2_O 6. If the maximum VT allowed by the anesthesia ventilator is achieved and the plateau pressure is lower than 30 cmH_2_O, increase the PEEP as needed, to a maximum of 20 cmH_2_O 7. Allow three breaths while maintaining plateau pressure of 30–40 cmH_2_O 8. Set VT, PEEP, respiratory rate, and I:E ratio back to pre-recruitment values
Lung re-expansion maneuver	1. Keep the non-ventilated under visual inspection, whenever possible 2. Connect the CPAP device with adequate oxygen flow (F_I_O_2_ 1.0) to the non-ventilated lung 3. Set CPAP to 10 cmH_2_O during 20 s 4. Set CPAP to 15 cmH_2_O during 20 s 5. Set CPAP to 20 cmH_2_O during 20 s 6. If performed as part of a rescue therapy for hypoxemia and oxygenation has been restored, reduce CPAP as soon as possible to 10 cmH_2_O, or 5 cmH_2_O, or disconnect the CPAP device

*RM* recruitment maneuver, *F*_*I*_*O*_*2*_ inspiratory fraction of oxygen, *I:E ratio* inspiratory to expiratory ratio, *VT* tidal volume, *PBW* predicted body weight, *PEEP* positive end-expiratory pressure, *CPAP* continuous positive airway pressure

A lung re-expansion maneuver of the non-ventilated lung may be necessary in both groups due to different reasons, including detection of air leaks by request of surgeons, as part of a rescue strategy due to hypoxemia, or before switching from OLV to TLV to re-expand the collapsed lung. Such a maneuver is performed in a hemodynamically stable patient (as judged by the anesthesiologist) and in agreement with the surgeon. To obtain standardization among centers, re-expansion maneuvers of non-ventilated lungs are performed with continuous positive airway pressure (Table 1).

#### Rescue strategies for intraoperative hypoxemia and intraoperative hypercapnia

If hypoxemia, defined as peripheral oxygen saturation (SpO_2_) < 90% for longer than 1 min occurs, rescue should be performed (Table 2). If hypercapnia (PaCO_2_ > 60 mmHg) with respiratory acidosis (pHa < 7.20) occurs during OLV, different steps are applied in the high and low PEEP groups (Table 2).

**Table 2:** Rescue strategies for intraoperative hypoxemia and hypercapnia

If hypoxemia occurs in the high PEEP group during TLV	1. Apply RM 2. Increase PEEP to 12 cmH_2_O and apply RM 3. Increase F_I_O_2_ in steps of 0.1 until 1.0 4. Consider stepwise decrease of PEEP down to 8 cmH_2_O
If hypoxemia occurs in the low PEEP group during TLV	1. Increase F_I_O_2_ in steps of 0.1 until 1.0 2. Apply RM 3. Increase PEEP to 6 cmH_2_O 4. Apply RM 5. Increase PEEP to 7 cmH_2_O 6. Apply RM
If hypoxemia occurs in the high PEEP group during OLV	1. Apply RM 2. Increase PEEP to 12 cmH_2_O and apply RM 3. Increase F_I_O_2_ in steps of 0.1 up to 1.0 4. Apply oxygen to the non-ventilated lung, consider using CPAP (see lung re-expansion maneuver) up to a pressure of 20 cmH_2_O, or selective oxygen insufflation via fiberscope 5. Consider stepwise decrease of PEEP of the ventilated lung to 8 cmH_2_O 6. Consider surgical intervention (e.g., clamping of the pulmonary artery by surgeon) 7. Consider administration of inhaled nitric oxide or prostacyclin, or intravenous almitrine (provided the drug is approved in your country/institution) 8. Switch to TLV
If hypoxemia occurs in the low PEEP group during OLV	1. Increase F_I_O_2_ in steps of 0.1 up to 1.0 2. Apply oxygen to the non-ventilated lung, consider CPAP therapy (re-expansion of the non-ventilated lung) up to a pressure of 20 cmH_2_O, or selective oxygen insufflation via fiberscope 3. Apply RM to the ventilated lung 4. Increase PEEP to 6 cmH_2_O 5. Apply RM to the ventilated lung 6. Increase PEEP to 7 cmH_2_O 7. Apply RM to the ventilated lung 8. Consider surgical intervention (e.g., clamping of the pulmonary artery by surgeon) 9. Consider administration of inhaled nitric oxide or prostacyclin, or intravenous almitrine (provided the drug is approved in your country/institution) 10. Switch to TLV
If hypercapnia (PaCO_2_ > 60 mmHg) with respiratory acidosis (pHa < 7.20) occurs during OLV, these steps are applied in the high and low PEEP groups	1. Increase the respiratory rate (maximum 30/min, while minimizing intrinsic PEEP) 2. Increase VT stepwise up to 7 mL/kg PBW 3. Switch to TLV

*RM* recruitment maneuver, *PEEP* positive end-expiratory pressure, *TLV* two lung ventilation, *F*_*I*_*O*_*2*_ inspiratory fraction of oxygen, *OLV* one lung ventilation, *CPAP* continuous positive airway pressure, *PaCO*_*2*_ arterial partial pressure of carbon dioxide, *pHa* arterial pH value, *VT* tidal volume, *PBW* predicted body weight

#### Standard procedures

To avoid interference with the trial intervention, routine elements of perioperative anesthesia care (including general anesthesia, postoperative pain management, physiotherapeutic procedures, and fluid management) are performed according to each center’s specific expertise and clinical routine. The following approaches are suggested (not mandatory) for anesthetic management:

- Use of inhaled isoflurane, desflurane or sevoflurane, intravenous propofol, remifentanil or sufentanil, and cisatracurium, atracurium, vecuronium, or rocuronium (as required)
- Use of sugammadex or a balanced solution of prostigmine, or neostigmine and atropine or glycopyrrolate for reversal of muscle relaxation, guided by neuromuscular function monitoring (for example, train-of-four stimulation)
- For postoperative pain management to achieve a VAS pain score below 3 use regional anesthesia, including epidural, paravertebral, and intercostal blockade, and consideration of indications, contra-indications, and local preferences is encouraged, but not obligatory
- Use of physiotherapy by early mobilization, deep breathing exercises with and without incentive spirometry, and stimulation of cough in the postoperative period
- Avoid fluid underload and overload
- Use of invasive measurement of arterial blood pressure whenever indicated
- Use of appropriate prophylactic antibiotics whenever indicated
- Use of gastric tubes, urinary bladder catheters, and more invasive monitoring according to individual needs, as well as local practice and/or guidelines

In addition, the study protocol stresses that routine intraoperative monitoring should include measurements of blood pressure, pulse oximetry, end-tidal carbon dioxide fraction, and electrocardiography. Every patient should receive at least one peripheral venous line to allow adequate fluid resuscitation during the study period. Other procedures should follow the Safe Surgery Checklist of the World Health Organization as published (www.who.int/patientsafety/safesurgery/en/index.html).

#### Minimization of bias

Allocation sequence is computer generated (nQuery Version 4.0) using permuted blocks with random sizes of 4, 6, and 8. Allocation is stratified per center with an allocation ratio of 1:1 for each group. The process of sequence generation and storage is managed by an independent database manager not involved in patient care. Randomization is then performed patient-by-patient using a web interface (REDcap™).

At each study site, at least two assessors are involved with the study. One assessor is involved with the intraoperative mechanical ventilation strategy and performs randomization as well as the interventions defined in the protocol. A second assessor, who is blinded to randomization, performs postoperative visits and assessment of primary and secondary endpoints.

#### Study endpoints

The primary endpoint is a collapsed composite of all PPC developing within the first 5 postoperative days. With this approach each complication has an equal weight. Patients who develop a least one complication are considered as meeting the primary endpoint.

PPC are defined as follows:

- aspiration pneumonitis (defined as respiratory failure after the inhalation of regurgitated gastric contents)
- moderate respiratory failure (SpO_2_ < 90% or PaO_2_ < 60 mmHg for 10 min in room air, responding to oxygen > 2 L/min)
- severe respiratory failure (need for non-invasive or invasive mechanical ventilation due to poor oxygenation)
- adult respiratory distress syndrome (mild, moderate, or severe according to the Berlin definition [24])
- pulmonary infection (defined as new or progressive radiographic infiltrate plus at least two of the following: antibiotic treatment, tympanic temperature > 38 °C, leukocytosis or leucopenia (white blood cell (WBC) count < 4000 cells/mm^3^ or > 12,000 cells/mm^3^) and/or purulent secretions)
- atelectasis (suggested by lung opacification with shift of the mediastinum, hilum, or hemidiaphragm towards the affected area, and compensatory over-inflation in the adjacent non-atelectatic lung)
- cardiopulmonary edema (defined as clinical signs of congestion, including dyspnea, edema, rales, and jugular venous distention, with the chest x-ray demonstrating increase in vascular markings and diffuse alveolar interstitial infiltrates)
- pleural effusion (chest x-ray demonstrating blunting of the costophrenic angle, loss of the sharp silhouette of the ipsilateral hemidiaphragm in upright position, evidence of displacement of adjacent anatomical structures, or (in supine position) a hazy opacity in one hemithorax with preserved vascular shadows)
- pneumothorax (defined as air in the pleural space with no vascular bed surrounding the visceral pleura)
- pulmonary infiltrates (chest x-ray demonstrating new monolateral or bilateral infiltrate without other clinical signs)
- prolonged air leakage (air leak requiring at least 7 days of postoperative chest tube drainage)
- purulent pleuritic (receiving antibiotics for a suspected infection, as far as not explained by the preoperative patient condition alone)
- pulmonary embolism (as documented by pulmonary arteriogram or autopsy, or supported by ventilation/perfusion radioisotope scans, or documented by echocardiography and receiving specific therapy)
- lung hemorrhage (bleeding through the chest tubes requiring reoperation, or three or more red blood cell packs)

Secondary clinical endpoints include:

- extended PPC, including bronchospasm (defined as newly detected expiratory wheezing treated with bronchodilators) or mild respiratory failure (SpO_2_ < 90% or PaO_2_ < 60 mmHg for 10 min in room air, responding to oxygen ≤ 2 L/min)
- intraoperative complications (use of continuous positive airway pressure for the non-ventilated lung, use of inhaled nitric oxide/prostacycline, use of selective fiberoscope insufflation, hypotension unresponsive to fluids and/or vasoactive drugs, new arrhythmias unresponsive to intervention, need for high dosage of vasoactive drugs (a dosage at the tolerance limit of the treating physician), need for massive transfusion, life-threatening surgical complication including major bleeding, tension pneumothorax, intracranial injury, hypoxemia and hypercapnia rescue maneuvers, deviation from prescribed PEEP or VT)
- postoperative extrapulmonary complications
- need for unexpected intensive care unit admission or readmission
- number of hospital-free days at day 28
- 90-day survival
- in-hospital survival
- arterial blood gas analysis during surgery (PaO_2_, PaCO_2_, pHa)
- any postoperative respiratory intervention (new requirement of non-invasive ventilation or mechanical ventilation)

Postoperative extrapulmonary complications include:

- systemic inflammatory response syndrome (presence of two or more of the following findings: body temperature < 36 °C or > 38 °C, heart rate > 90 beats per minute, respiratory rate > 20 breaths per minute or, on blood gas, a PaCO_2_ < 32 mmHg (4.3 kPa), WBC count < 4000 cells/mm^3^ or > 12,000 cells/mm^3^, or > 10% band forms)
- sepsis (systemic inflammatory response syndrome in response to a confirmed infectious process; infection can be suspected or proven (by culture, stain, or polymerase chain reaction), or a clinical syndrome pathognomonic for infection)
- specific evidence for infection includes WBCs in normally sterile fluid (such as urine or cerebrospinal fluid, evidence of a perforated viscera (free air on abdominal x-ray or computer tomography scan, signs of acute peritonitis), abnormal chest x-ray consistent with pneumonia (with focal opacification), or petechiae, purpura, or purpura fulminans)
- severe sepsis (sepsis with organ dysfunction, hypoperfusion, or hypotension), septic shock (sepsis with refractory arterial hypotension or hypoperfusion abnormalities in spite of adequate fluid resuscitation); signs of systemic hypoperfusion may be either end-organ dysfunction or serum lactate greater than 4 mmol/dL, other signs include oliguria and altered mental status
- septic shock id defined as sepsis plus hypotension after aggressive fluid resuscitation, typically upwards of 6 L or 40 mL/kg of crystalloid
- extra-pulmonary infection (wound infection + any other infection)
- coma (Glasgow Coma Score < 8 in the absence of therapeutic coma or sedation)
- acute myocardial infarction (detection of rise and/or fall of cardiac markers (preferably troponin) with at least one value above the 99th percentile of the upper reference limit, together with symptoms of ischemia, electrocardiography changes indicative of new ischemia, development of pathological Q-waves, or imaging evidence of new loss of viable myocardium or new regional wall motion abnormality or sudden unexpected cardiac death, involving cardiac arrest with symptoms suggestive of cardiac ischemia (but death occurring before the appearance of cardiac markers in blood))
- acute renal failure (renal failure documented as follows: Risk: increased creatinine × 1.5 or glomerular filtration rate (GFR) decrease > 25% or urine output (UO) < 0.5 mL/kg/h × 6 h; Injury: increased creatinine × 2 or GFR decrease > 50% or UO < 0.5 mL/kg/h × 12 h; Failure: increased creatinine × 3 or GFR decrease > 75% or UO < 0.3 mL/kg/h × 24 h or anuria × 12 h; Loss: persistent acute renal failure = complete loss of kidney function > 4 weeks)
- disseminated intravascular coagulation (score documented as follows: platelet count < 50 (2 points), < 100 (1 point), or ≥ 100 (0 points); D-dimer > 4 μg/mL (2 points), > 0.39 μg/mL (1 point) or ≤ 0.39 μg/mL (0 points); prothrombin time > 20.5 s (2 points), > 17.5 s (1 point), or ≤ 17.5 s (0 points), if ≥ 5 points: overt disseminated intravascular coagulation)
- stroke (new clinical signs of stroke lasting longer than 24 h and corresponding findings in radiologic imaging)
- hepatic failure (hepatic failure during short-term follow-up (5 postoperative days) is considered as follows: bilirubin serum level > 2 mg/dL + elevation of alanine amino transferase/aspartate amino transferase + lactate dehydrogenase × 2 above normal values; during long-term follow-up (until postoperative day 90) at new presence of hepatic encephalopathy and coagulopathy (international normalized ratio (INR) > 1.5) within 8 weeks after initial signs of liver injury (e.g., jaundice) without evidence for chronic liver disease)
- gastrointestinal failure (any type of gastrointestinal bleeding or gastrointestinal failure score documented as follows: 0 = normal gastrointestinal function; 1 = enteral feeding with under 50% of calculated needs or no feeding 3 days after abdominal surgery; 2 = food intolerance or intra-abdominal hypertension; 3 = food intolerance and intra-abdominal hypertension; and 4 = abdominal compartment syndrome)

At the discretion of participating centers, blood and urine samples are collected preoperatively as well as directly postoperative and on the postoperative days 1–5. Samples will be analyzed centrally for systemic markers of inflammation and coagulation (including but not limited to interleukins 6 and 8, thrombin-antithrombin, protein C, and plasminogen activator inhibitor-1) as well as systemic markers of injury to the lungs (including but not limited to plasma E-cadherin, soluble receptor for advanced glycation end-products, surfactant proteins A and D, and distal organs, including renal injury (including but not limited to plasma/urine neutrophil gelatinase-associated lipocalin, and cystatin C). The standard operating procedure for collecting and processing plasma and urine is available in Additional file 1.

#### Study visits and data collection

**Fig. 3:**
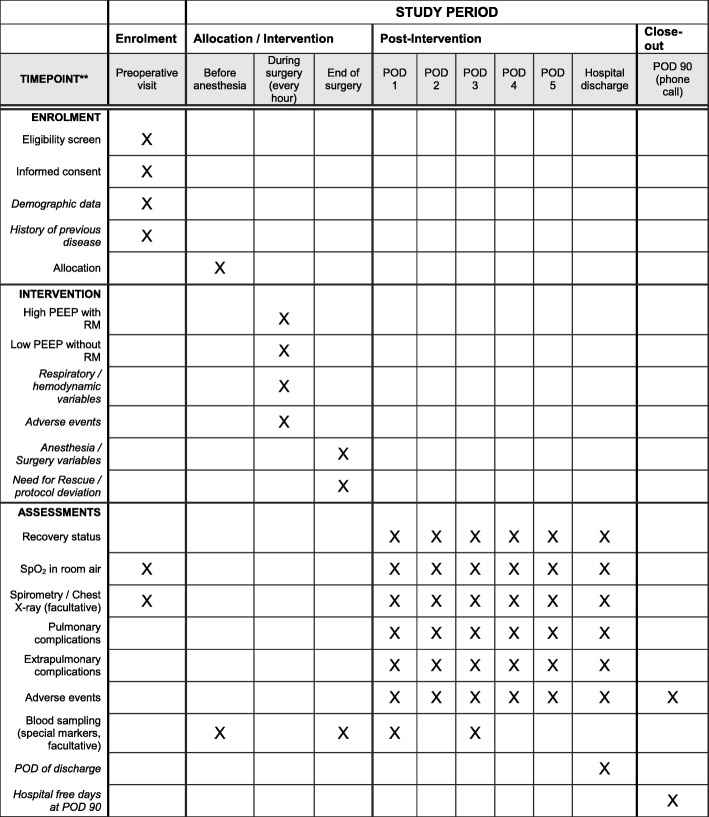
Schedule of enrolment, interventions, and assessments. *POD* postoperative day, *PEEP* positive end-expiratory airway pressure, *RM* (lung) recruitment maneuver, *SpO*_*2*_ peripheral oxygen saturation

Patients are visited preoperatively, intraoperatively, daily between postoperative days 1 and 5, and on discharge. On postoperative day 90, patients are contacted by phone (Fig. 3).

Patients are screened according to inclusion criteria. All patients meeting the inclusion criteria are registered in a screening log file by each center. Eligible patients meeting none of the exclusion criteria are asked by the physician for written informed consent (the consent form and information to study patients form are available in Additional file 1).

Baseline variables are collected, including gender, age, height, weight, ARISCAT Score, physical status according to the American Society of Anesthesiologists, functional status according to cumulated ambulation score, metabolic equivalents, cardiovascular status (heart failure according to the New York Heart Association, coronary heart disease according to Canadian Cardiovascular Society, atrial flutter/fibrillation, arterial hypertension), pulmonary status (chronic obstructive pulmonary disease, including steroids and/or inhalation therapy use, respiratory infection within the last month, use of non-invasive ventilatory support), history of obstructive sleep apnea (including Apnea and Hypopnea index or STOP-Bang score in patients without diagnosis of obstructive sleep apnea), metabolic status (diabetes mellitus, including data on treatment), history of active cancer, smoking status, alcohol status, gastroesophageal reflux, oral medication (e.g., use of antibiotics, statins, aspirin), preoperative organ function (SpO_2_ in supine position, upper body elevated 30–45 degrees breathing room air; if possible, respiratory rate, heart rate, mean arterial pressure, body temperature, airway secretion, including data on purulence, visual analogue scales (1–10) for dyspnea, thoracic rest pain and coughing pain).

Preoperative non-mandatory measurements include spirometry (arterial partial pressure of oxygen, carbon dioxide and pH value, forced vital capacity (FVC), forced expiratory volume in one second (FEV_1_), Tiffeneau value (FEV_1_/FVC), total lung capacity, diffusing capacity for carbon monoxide, and maximal oxygen consumption), predicted postoperative respiratory function (predicted postoperative FVC, FEV_1_, and diffusing capacity for carbon monoxide), chest x-ray (assessed for infiltrates, pleural effusion, atelectasis, pneumothorax, and cardiopulmonary edema) as well as routine laboratory tests (including hemoglobin, hematocrit, WBC count, platelet count, INR, partial thromboplastin time, creatinine, blood urea nitrogen, alanine amino transferase, aspartate amino transferase, bilirubin, c-reactive protein, and procalcitonin).

During the intraoperative visit, both surgery- as well as anesthesia-related data are recorded, including duration of anesthesia (from intubation to extubation or exit of operating room if on mechanical ventilation), duration of OLV and TLV, duration of surgery (from incision to closure), total blood loss, total urine output, side of OLV and side of surgery, method of lung separation (double lumen tube, endobronchial blocker, double lumen tube with embedded camera), way of placement confirmation (fiberoptic bronchoscopy, embedded camera), administration of antibiotics, use of regional anesthesia (epidural, paravertebral, other), use of non-invasive ventilation during induction, patient position during induction, patient temperature at the end of surgery, monitoring of neuromuscular function during anesthesia, use of neuromuscular blocker antagonists, priority and type of surgery, wound classification, type of surgical resection, patient position during surgery, estimated amount of lung resection, and drugs and fluids administered during anesthesia (e.g., anesthetics, vasoactive drugs, transfusion).

Ventilator settings, hemodynamics, need for rescue strategy, and adverse events (AEs) are recorded at anesthesia induction, with the patient in final surgical position and TLV, 10 min after OLV, hourly thereafter during OLV, and at the end of surgery with TLV in supine position. The routine measurements are documented first, then the gas probes are taken; thereafter, the RM is performed in the high PEEP group.

RM are documented during the plateau phase of the RM in the high PEEP group after bronchoscopy or disconnection of the ventilated lung from the mechanical ventilator, after the beginning of OLV, every 1 hour during OLV, after re-expansion of the non-dependent lung and resumption of TLV, and at the end of surgery in supine position.

Clinical data, including actual organ function and the presence of PPC, are scored during postoperative visits on a daily basis. Additionally, secondary endpoints, such as postoperative extrapulmonary complications, need for unexpected intensive care unit admission or readmission, and any type of postoperative respiratory intervention, are recorded. On day 1 after surgery, fluid and transfusion data are recorded in a detailed manner. Furthermore, the use of physiotherapy, breathing exercises, antibiotics as well as the cumulated ambulation score, status of wound healing, postoperative nausea, and vomiting are assessed.

Non-mandatory measures include chest x-ray, spirometry, and routine laboratory tests. Patients will be visited until discharge.

The number of hospital-free days at day 28 (including readmission since hospital discharge) and 90-day survival are calculated. Day 90 is defined as the last day of follow-up; accordingly, patients still admitted to hospital will be last visited on that day.

#### Study dropouts

Participation in the trial is voluntary. Patients have the right to withdraw consent to the study at any time for any reason without any consequence for further medical treatment. The reasons and circumstances for study discontinuation will be documented in the case report form (CRF). Primarily, all data will be analyzed according to the intention-to-treat principle. Secondarily, data will be analyzed per-protocol.

#### Handling of data

The objective of the clinical data management plan is to provide high-quality data by adopting standardized procedures to minimize the number of errors and missing data and, consequently, to generate an accurate database for analysis. Two members of the research team perform study monitoring. Remote monitoring is performed to signal early aberrant patterns, issues with consistency, credibility, and other anomalies. On-site assessment of protocol adherence and completeness of the research dossier will be conducted in up to 10 sites including the highest number of patients, and also neighbor sites to them.

Patient data are collected in pseudonymous form using a patient (identification) number composed of six digits, the first three of which correspond to the site ID and the remaining digits correspond to the patient inclusion number at the respective site. Study data are collected and managed using REDCap™ electronic data capture tools hosted at the Clinical Trial Coordination Center (KKS) of the University of Dresden, Germany. REDCap™ (Research Electronic Data Capture) is a Secure Sockets Layer encrypted, password-protected, web-based application designed to support data capture for research studies [25]. Full access to the final trial dataset will be granted to selected investigators only. If a sub-study is approved by the steering committee, access only to data related to the sub-study will be granted to the respective principal investigator.

#### Sample size calculations

**Fig. 4:**
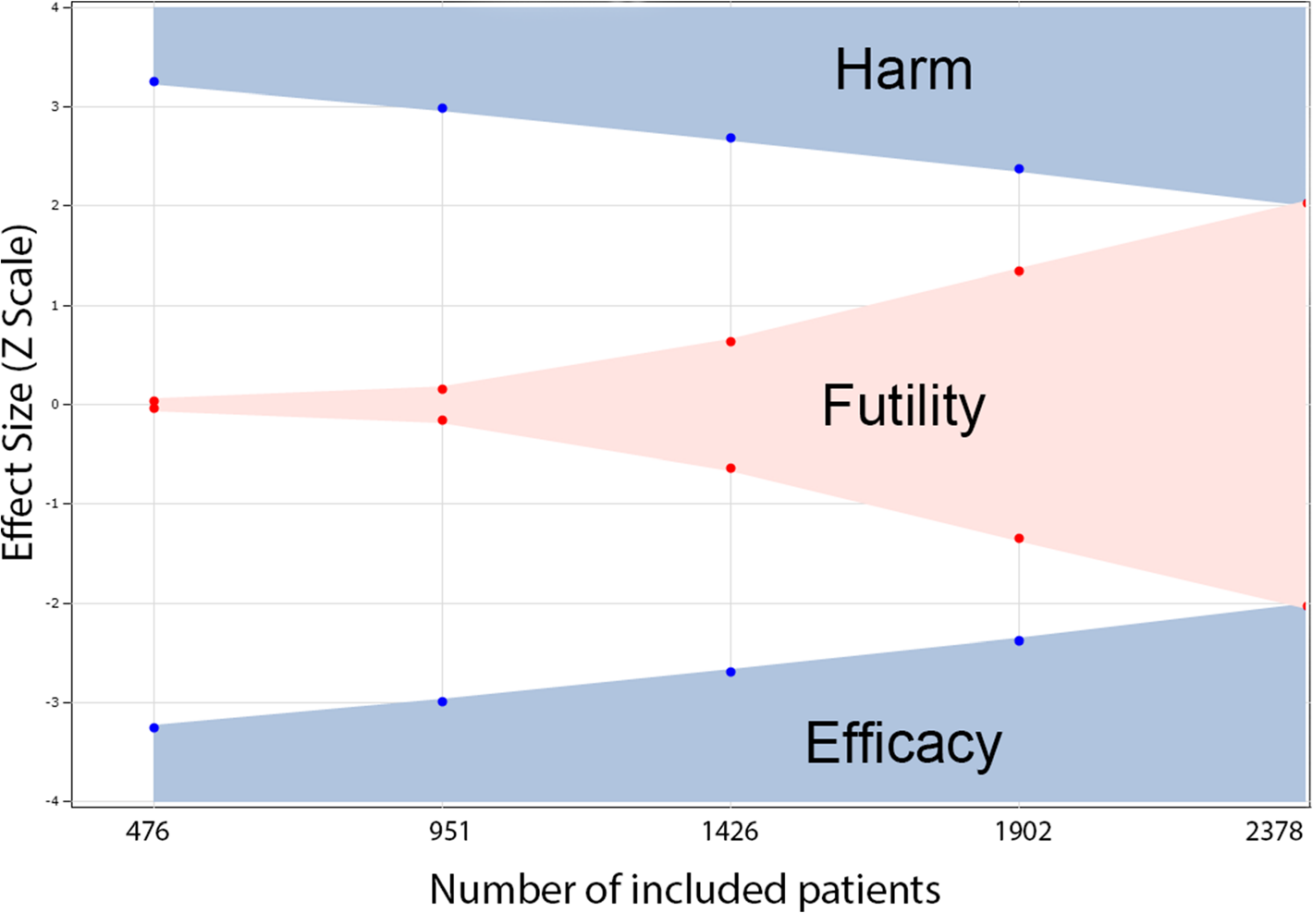
Effect size (Z) according to enrollment of patients in the PROTHOR trial (including dropouts). Values of Z were obtained from an adaptive sequential design (see text) with stopping criteria for harm, futility, and efficacy of the intervention

For this trial, we have planned to use an adaptive trial design, which accumulates data and uses external information to modify aspects of the design without undermining the validity and integrity of the trial. The group sequential methods design gives us the possibility for early stopping of the study if the experimental treatment shows a statistically significant therapeutic advantage at an interim assessment, but also allows early stopping for futility if the interim analysis reveals that, with high probability, the trial will be negative (Fig. 4).

Sample size calculation was based on our primary study endpoint, taking data collected from a subset of patients undergoing OLV for thoracic surgery in a prospective observational, multicenter, international study (LAS VEGAS) [26] into account. LAS VEGAS showed an incidence of approximately 23% for a PPC composite comparable to the present definition. Assuming a significance level of 0.05 and a power of 90% to detect the expected difference in postoperative pulmonary complications between the high PEEP group of 17.25% and the low PEEP group of 23% (risk ratio of 0.75), a sample size of 2259 has been calculated. Assuming a dropout rate of 5%, a total of 2378 patients have to be included in the study.

We used the software package East^®^ for sample size calculations (East^®^, Version 6.3.1, Cytel Inc., USA). The Difference of Proportions test has been used to compare the independent samples from two populations (Group Sequential Design for a Binomial Superiority Trial, discrete endpoint two sample test, parallel design, difference of proportions, using the unpooled estimate of variance). The sample size calculation was done with the following parameters: Superiority Design, two-sided test; alpha 0.05; Power 0.9, allocation ratio 1; Proportion_1_ = 0.23; Proportion_2_ = 0.1725; Difference in Proportions = − 0.058.

We used an alpha-spending function to generate efficacy boundaries and a beta-spending function to generate futility boundaries (Fig. 4; gamma family spending function, type I error 0.05, type II error 0.1). By using a gamma of − 4 for the alpha and gamma of − 2 for the beta spending function we have a moderate hurdle for early stopping for efficacy and a reasonable chance to stop early due to futility (Table 3).

**Table 3:** Z-statistic boundaries and boundary crossing probabilities

Look	Information fraction	*N*	Cumulative alpha spent	Cumulative beta spent	Z-efficacy/harm	Z-futility	Boundary crossing probabilities under H1	
							Efficacy	Futility
1	0.2	452	0.001	0.008	±3.252	±0.031	0.042	0.008
2	0.4	904	0.004	0.019	±2.986	±0.152	0.17	0.011
3	0.6	1355	0.009	0.036	±2.692	±0.631	0.281	0.017
4	0.8	1807	0.022	0.062	±2.374	±1.344	0.263	0.026
5	1.0	2259	0.05	0.1	±2.025	±2.025	0.143	0.038

Values were calculated using power = 0.90, alpha = 0.05, gamma spending function − 4 for the alpha and − 2 for the beta, expected incidence of postoperative pulmonary complications of 23% and 17.25% in the lower and higher positive end-expiratory pressure groups, respectively. Number of patients (*N*) is shown without correcting for dropouts. Look, interim analysis; H1, hypothesis 1 (group difference exists)

We constructed a non-binding futility boundary in such a way that it can be overruled if desired without inflating the type 1 error. This flexibility is important, since the data monitoring committee might well prefer to keep the trial going to gather additional information, despite crossing the futility boundary.

We planned to take five interim assessments at the data for evidence of efficacy, harm, and/or futility with the aim of possibly stopping the trial early. The planned number of assessments describes the number of time points, including the closing date of the study, at which the investigator plans to analyze the thus far collected data. The spacing of assessments will be equal. Therefore, interim analyses will be performed after 20% (476 patients), 40% (952 patients), 60% (1426 patients), 80% (1902 patients), and 100% of patients (2378 in total) included.

Patients will be randomly assigned to one of the two groups using a website-based data entry and randomization platform (REDcap™, Ver 6.6.2 Vanderbilt University, Tennessee, USA). Randomization will be conducted using blocks of 4, 6, and 8 patients, in aleatory fashion. Thereby, group sizes will be comparable at interim analyses, which will be conducted in a group-blinded manner.

#### Statistical analysis

Continuous distribution of the data will be assessed by visual inspection of histograms and D’Agostino–Pearson’s normality tests. For both arms, the baseline characteristics will be expressed as counts and percentages, means and standard deviations, or medians and interquartile ranges whenever appropriate.

Ventilatory parameters and vital signs over the surgery will be analyzed using a mixed-effect model with repeated measures and with patients and centers as a random-effect. No or minimal losses to follow-up for the primary and secondary outcomes are anticipated. Complete-case analysis will be carried out for all the outcomes. However, if more than 1% of missing data were found for the primary outcome, a sensitivity analysis using multiple imputations and estimating equation methods will be carried out.

Hypothesis tests will be two-sided with a significance level of 5% with exception of the primary outcome, due to the correction for the interim analyses. We will not adjust *p* values for multiple comparisons. Analyses will be performed using the R (R Core Team, 2016, Vienna, Austria) program.

##### Primary outcome

The effects of the intervention on incidence of PPC will be reported as numbers and percentages and estimated with risk ratios and 95% confidence intervals calculated with Wald’s likelihood ratio approximation test and with χ^2^ tests for hypothesis testing. For the analysis of the primary outcome, the result will be considered significant if the *p* value is less than 0.0428 (correspondent to the Z-value of 2.025 for efficacy or futility in the final analysis in Table 3). Kaplan–Meier curves will be used to report time to PPC. Curves will be compared with the log-rank tests, and hazard ratios with 95% confidence intervals will be calculated with Cox proportional hazard models without adjustment for covariates. The proportional hazard assumptions will be tested using scaled Schoenfeld residuals and alternative parametric survival models will be used if the proportionality assumption is not sustained.

##### Secondary outcomes

The effect of the intervention on secondary binary outcomes will be assessed with risk ratio and 95% confidence intervals calculated with Wald’s likelihood ratio approximation test and with χ^2^ tests for hypothesis testing. The effects of the intervention on hospital-free days at day 28 will be estimated with a Student *t* test and reported as the mean difference between the two groups. The consistency of the findings of the Student *t-*test for the hospital-free days at day 28 will be confirmed according to the mean ratio calculated by a generalized additive model considering a zero-inflated beta distribution.

Finally, 90-day mortality will be assessed using Kaplan–Meier curves, and hazard ratios with 95% confidence intervals will be calculated with Cox proportional hazard models without adjustment for covariates. The proportional hazard assumptions will be tested using scaled Schoenfeld residuals and alternative parametric survival models will be used if the proportionality assumption is not sustained.

##### Subgroup analyses

Treatment effects on incidence of PPC will be analyzed according to the following subgroups: (1) non-thoracoscopic versus thoracoscopic; (2) lateral decubitus versus supine position; (3) baseline SpO_2_ < 96% versus SpO_2_ ≥ 96%; and (4) COPD versus non-COPD. The effects on subgroups will be evaluated according to the interaction effects between each subgroup and the study arms by generalized linear models and presented in a forest plot.

Per-protocol analyses: The per-protocol population will consist of patients truly ventilated with the pre-specified protocol. Thus, patients will be excluded from this population if receiving PEEP < 10 cmH_2_O in the high PEEP group or PEEP > 5 cmH_2_O and F_I_O_2_ < 1.0 in the low PEEP group, in any measurement during the surgery.

##### Other exploratory analyses

As a sensitivity analysis, the effect of the intervention on the primary outcome will be re-estimated using a generalized linear mixed-effect model with stratification variables (center) as random effects. Since the primary outcome of the present study is a composite one, the choice of the statistical method is an important part of the design because various methods provide different power, depending on the situation. In addition to the standard analysis described above, the following analyses will be performed:

- Count analysis – the number of positive component events (i.e., ‘count’) across the composite will be assessed. The groups will be compared on the count using a Mann–Whitney test, and the odds ratio with the 95% confidence interval will be assessed with a proportional odds logistic regression model
- Individual component analysis – the effect of the intervention in each component will be analyzed using a generalized linear model using a Bonferroni correction for multiple comparisons; the 99.64% Bonferroni-corrected confidence intervals will be reported (1 – 0.05/14 = 0.9964)
- Common effect test – a multivariate (i.e., multiple outcomes per subject) generalized estimating equations (GEE) model will be used to estimate a common effect odds ratio across the components
- Average relative effect test – the average relative effect test will be assessed by averaging the component-specific treatment effect from the distinct effects model, and testing whether the average is equal to zero; in the GEE distinct effect model, a distinct treatment effect is estimated for each component
- Heterogeneity of treatment effect – heterogeneity of treatment effect across components will be assessed by a treatment-by-component interaction test in the distinct effects GEE model
- Clinical severity weight – each component will be weighted by a clinical severity weight determined a posteriori; a multivariate (i.e., multiple outcomes per subject) GEE model will be used to estimate a common effect odds ratio across the components while applying the severity weights

##### Cleaning and locking of the database

The database will be locked as soon as all data are entered and all discrepant or missing data are resolved – or if all efforts are employed and we consider that the remaining issues cannot be fixed. In this step, the data will be reviewed before database locking. After that, the study database will be locked and exported for statistical analysis. At this stage, permission for access to the database will be removed for all investigators, and the database will be archived.

##### Missing data

No or minimal losses to follow-up for the primary and secondary outcomes are anticipated. Complete-case analysis will be carried out for all the outcomes, that is, excluding patients with missing data in the outcome of interest. However, if more than 1% of missing data were found for the primary outcome, a sensitivity analysis using multiple imputations and estimating equation methods will be performed.

#### Sub-studies

Participating centers are allowed to conduct sub-studies provided that (1) no interference with the primary protocol occurs; (2) approval by the local institutional review board is obtained; and (3) the steering committee accepts the proposal according to its originality, feasibility, and importance. Publication of sub-studies, in any form, is strictly forbidden until the results of the primary study have been published.

### Trial organization

The trial is managed by a team consisting of the chief investigator (Mert Sentürk), the trial coordinator (Thomas Kiss), the statisticians (A. Serpa Neto, K. Schubert and M. Kuhn), the informatics technician responsible for the web-based electronic data capture system (Marko Kaeppler), and independent monitors. A steering committee contributed to the design and revision of the study, and will be responsible for interpretation of data and compilation of a resulting manuscript.

Patient data and safety is closely monitored by a data safety and monitoring board (DSMB) that consists of a chairperson (Daniel Sessler) and four further members (Arthur Slutsky, Andreas Hoeft, Jean-Louis Vincent, Jennifer Hunter). All AEs entered into the electronic CRF within pre-specified time frames, including severe AEs and suspected unexpected severe adverse reactions, are monitored by an international AE manager (Ary Serpa Neto), who provides the DSMB with reports for review. The DSMB further monitors the overall status of the trial, e.g., progress of patient enrollment, general adherence to protocol, and completeness of data entry. Monitoring visits will be conducted as deemed necessary by the DSMB.

National coordinators are responsible for administration and communication with local principal investigators, as well as assistance during trial management and data collection.

When submitting the report on the results of the trial for possible publication, sites will be eligible to one collaborative co-authorship plus a further co-authorship for every 20 treated patients with complete datasets.

## Discussion

The PROTHOR trial was designed to determine whether a high level of PEEP with RM, as compared to low PEEP without RM, during OLV for thoracic surgery, prevents PPC. We opted for testing the impact of two ventilation strategies at the same low VT in order to focus on the independent effects of different airway pressures, especially PEEP.

The decision to use a PEEP value of 5 cmH_2_O in the low PEEP group has been derived from a recent study on the practice of intraoperative mechanical ventilation and consensus agreement of the steering committee [26]. In order to allow generalizability of results and to impact on clinical practice, we opted for a pragmatic study, where a fixed level of high PEEP is used. The decision of using a PEEP of 10 cmH_2_O in the high PEEP group was based on the fact that this value, on average, resulted in maximal dynamic compliance of the respiratory system during OLV in a recent study, and was accompanied by minor variability only [27]. Additionally, this value is only 2 cmH_2_O higher than needed to effectively increase oxygenation and decrease physiological dead space [21, 28], while avoiding substantial hemodynamic impairment.

Even a PEEP titrated to a respiratory mechanics target, for example, the compliance of the respiratory system [27], represents a compromise in terms of regional overdistension and collapse-reopening of lung units. Depending on regional differences, even this optimal PEEP will not completely prevent atelectasis formation [29]. Thus, even an individualized PEEP titration in the high PEEP group would also result in a compromise between atelectrauma and volutrauma or barotrauma, and likely not differ importantly from the value selected a priori in the present trial.

The RM is based on a stepwise increase of VT and PEEP. This maneuver allows opening of lung units without interruption of mechanical ventilation and ensures standardization across different centers. Since it uses volume-controlled ventilation, virtually all anesthesia ventilators can perform this maneuver. The target airway pressure range for recruitment was based on the fact that a level of 30 cmH_2_O was proposed in a recent study [30], and that airway pressure exceeding 40 cmH_2_O does not importantly contribute to open lungs even in mild acute respiratory distress syndrome [31].

We decided for a combination of RM and PEEP in the high PEEP group. PEEP per se may not be enough to open atelectatic lung units. A CT study showed that, in patients at higher risk for development of intraoperative atelectasis, the combination of high PEEP and RM was able to revert lung collapse, whereas isolated high PEEP or RM did not achieve the same effect [32]. Furthermore, during OLV, RM followed by PEEP has been shown to be associated with a more homogenous distribution of ventilation [33].

The inspiratory time of approximately 5 s was chosen to allow enough pressure versus time product (over at least three consecutive cycles) to open atelectatic lung units. We opted for recruiting lungs not only after intubation, but also every hour thereafter, in order to revert possible progressive de-recruitment at PEEP of 10 cmH_2_O. For both the lower and higher PEEP groups, rescue protocols for the progression of intraoperative hypoxemia were defined in order to protect patients while allowing a standardized approach that minimizes the interference with the respective interventions. Importantly, deviations of the protocol, even rescue due to hypoxemia, are explicitly allowed, provided this in the best interest of patients.

It is worth noting that recommendations have been made also with regard to different phases and aspects of the anesthetic procedure, including monitoring, choice of anesthetics agents, muscle paralysis and its reversal, intravascular volume loading and maintenance, and postoperative analgesia. However, PROTHOR is a pragmatic study and influence on local practice of respective sites is kept at a minimum, focusing on factors that are more directly related with the hypothesis investigated.

Besides postoperative respiratory failure, several other adverse pulmonary events seem to add to the odds of mortality in the surgical population. In-hospital length of stay and mortality increase with the number of single pulmonary AEs in the postoperative period [3]. Therefore, in the PROTHOR trial we opted for a binary collapsed composite of single adverse pulmonary events as primary endpoint, despite the fact that single events may differ in terms of severity. Thus, the use of PPC as primary endpoint in the PROTHOR trial not only has clinical relevance for the practicing anesthetist, but increases the study power due to summation of incidences of single AEs. In spite of this, the study analysis will address not only the composite itself, but also the incidence of each element separately.

Not only the respiratory but also other organ systems may be impaired in the postoperative period in thoracic surgery patients. Thus, the analysis will also address the impact of intraoperative mechanical ventilation on single organs and a collapsed composite of non-pulmonary AEs, namely postoperative extrapulmonary complications. In addition, further relevant outcome measures that might be related to PPC and postoperative extrapulmonary complications, especially the hospital-free days at day 28, will be addressed. This outcome variable is not only a measure of morbidity, but also has direct impact on related health costs. Since we anticipate that, during surgery, both the lower and the higher PEEP groups will impact on intraoperative oxygenation, respiratory system mechanics, and arterial blood pressure, intraoperative respiratory function and hemodynamic variables will also be evaluated.

Much attention has been paid to safety in the PROTHOR trial. Accordingly, data and patient safety during the PROTHOR trial is closely monitored by a DSMB. Additionally, an AE manager has been designated. A web-based electronic data capture system (REDCap™) is used for building the database within a secure system, while allowing access to the eCRF and randomization of patients into groups.

We included complications that may be not directly related to VILI, more specifically pulmonary embolism and lung hemorrhage. However, the mechanical ventilation setting has been identified as an independent risk factor for venous thromboembolism [34]. Both mechanical ventilation and PEEP tend to decrease right and left ventricular preload, especially in the presence of hypovolemia and may increase venous thromboembolism risk by exacerbation of venous stasis. Recruitment maneuvers but also redistribution of lung perfusion during OLV and TLV may facilitate lung hemorrhage, which has been defined as bleeding through the chest tubes requiring reoperation or transfusion.

In summary, PROTHOR is the first randomized controlled trial in patients undergoing thoracic surgery that is adequately powered to compare the effects of intraoperative high PEEP with RM versus low PEEP without RM during OLV on PPC. The results of the PROTHOR trial will support anesthesiologists in their decision to set intraoperative PEEP during OLV with low VT for thoracic surgery.

### Trial status

The PROTHOR trial is currently recruiting patients. Recruitment started January 2017. Estimated completion date 2021.

**Table:** 

Site name	Collaborator surname	Collaborator name	Email address
Military Medical Academy, Belgrade, Serbia	Neskovic	Vojislava	vojkan43@gmail.com
	Radovic	Nevena	nevence1@yahoo.com
	Rondovic	Goran	grondovic@gmail.com
	Stamenkovic	Dusica	dusicastamenkovic@yahoo.com
	Vukovic	Rade	radvuk@gmail.com
	Zeba	Snjezana	snjezanazeba@hotmail.com
Department of Anaesthesiology, University Hospital Aachen, Aachen, Germany	Rossaint	Rolf	rrossaint@ukaachen.de
	Coburn	Mark	mcoburn@ukaachen.de
	Kowark	Ana	akowark@ukaachen.de
	Ziemann	Sebastian	sziemann@ukaachen.de
	van Waesberghe	Julia	jvanwaesberg@ukaachen.de
Department of Anesthesiology, Academic Medical Center Amsterdam, Amsterdam, The Netherlands	Bauer	Wolfgang	w.o.bauer@amc.uva.nl
	Terwindt	Lotte	l.e.terwindt@amc.uva.nl
Attikon University Hospital, Athens, Greece	Kostopanagiotou	Kostas	kostop@hotmail.co.uk
	Kostroglou	Andreas	andreaskostr@gmail.com
	Kyttari	Katerina	akyttari@gmail.com
	Sidiropoulou	Tatiana	tatianasid@gmail.com
University Hospital Clínic de Barcelona, Spain	Jiménez Andújar	María-José	jjimenez@somclinic.cat
	López-Baamonde	Manuel	lopez10@clinic.cat
	Navarro Ripoll	Ricard	rnavarr1@clinic.cat
	Rivera Vallejo	Lorena	lorivera@clinic.cat
Weill Cornell Medicine, Department of Anesthesiology, New York, USA	Henry	Matthew	mah2065@med.cornell.edu
	Jegarl	Anita	anj2024@med.cornell.edu
	Murrell	Matthew	mtm9006@med.cornell.edu
	O’Hara	Patrick	pao2011@med.cornell.edu
	Steinkamp	Michele	mls9004@med.cornell.edu
Fachkrankenhaus Coswig GmbH Zentrum für Pneumologie, Allergologie, Beatmungsmedizin, Thoraxchirurgie	Kraßler	Jens	krasslerj@fachkrankenhaus-coswig.de
	Schäfer	Susanne	schaefers@fachkrankenhaus-coswig.de
Department of Anesthesiology and Intensive Care Medicine, Pulmonary Engineering Group, University Hospital Carl Gustav Carus, Dresden, Germany	Becker	Charlotte	charlotte-becker@gmx.net
	Birr	Katja	katjabirr@gmail.com
	Bluth	Thomas	thomas.bluth@uniklinikum-dresden.de
	Gama de Abreu	Marcelo	mgabreu@uniklinikum-dresden.de
	Hattenhauer	Sara	sara.hattenhauer@uniklinikum-dresden.de
	Kiss	Thomas	thomas.kiss@uniklinikum-dresden.de
	Scharffenberg	Martin	martin.scharffenberg@uniklinikum-dresden.de
	Teichmann	Robert	teichmannrobert@aol.com
	Wittenstein	Jakob	jakob.wittenstein@uniklinikum-dresden.de
Department of Morpholo gy, Surgery and Experimental Medicine, University of Ferrara, Ferrara, Italy	Costanza	Vitali	costanza.vitali@student.unife.it
	Savino	Spadaro	savinospadaro@gmail.com
	Volta	Carlo Alberto	vlc@unife.it
	Ragazzi	Riccardo	rgc@unife.it
	Calandra	Camilla	camilla.calandra@gmail.com
Dept of Anesthesia and Intensive Care, University of Foggia, Italy, OO Riuniti Hospital			
	Mariano	Karim	karim_mariano@hotmail.it
	Mirabella	Lucia	lucia.mirabella@unifg.it
	Mollica	Giuseppina	giusymollica@virgilio.it
	Montrano	luigi	luigi.montrano@unifg.it
Department of Anesthesiology and Intensive Care Medicine Clinic, Medical Center - University of Freiburg, Faculty of Medicine, University of Freiburg, Germany	Loop	Torsten	torsten.loop@uniklinik-freiburg.de
	Semmelmann	Axel	axel.semmelmann@uniklinik-freiburg.de
	Wirth	Steffen	steffen.wirth@uniklinik-freiburg.de
Department of Anesthesiology, Fudan University Shanghai Cancer Center; Department of Oncology, Shanghai Medical College, Fudan University, Shanghai, China	Miao	Changhong	miaochh@aliyun.com
	Zhong	Jing	ziteng1934@163.com
	Lv	Hu	lvhu086@126.com
	Wang	Hui	2502425738@qq.com
	Zhang	Xue	zx02190554@126.com
	Zhang	Yue	aileencheung0807@163.com
IRCCS San Martino Policlinico Hospital, Genoa, Italy	Pelosi	Paolo	ppelosi@hotmail.com
	Corsi	Laura	corsilaura@yahoo.it
	Partroniti	Nicolò	nicoloantonino.patroniti@unige.it
	Mandelli	Maura	maura.mandelli@gmail.com
	Bonatti	Giulia	giulia.bonatti@gmail.com
	Simonassi	Francesca	francesca.simonassi@gmail.com
	Gratarola	Angelo	a.gratarola@gmail.com
Insular Hospital, Gran Canaria, Spain	Rodriguez Ruiz	Juan José	juanjo.rodriguezruiz@gmail.com
	Socorro	Tania	austania@gmail.com
University Hospital of Heraklion, Heraklion, Greece	Christofaki	Maria	mchristofaki@yahoo.gr
	Nyktari	Vasileia	vnyktari@gmail.com
	Papaioannou	Alexandra	papaioaa@uoc.gr
University Istanbul University, Istanbul Medical Faculty, Department of Anaesthesiology and Intensive Care, Istanbul, Turkey	Şentürk	Nüzhet Mert	senturkm@istanbul.edu.tr
	Bingul	Emre	dremrebingul@gmail.com
	Orhan Sungur	Mukadder	mukadder.orhan@gmail.com
	Sungur	Zerrin	zerrin_sr@yahoo.com
University Hospital of Munich, Munich, Germany	Heidegger	Manuel	manuel.heidegger@campus.lmu.de
	Dossow	Vera	vera.dossow@med.uni-muenchen.de
	Jerichow	Wiebke	w.jerichow@web.de
	Kammerer	Tobias	tobias.kammerer@med.uni-muenchen.de
	Richter	Julia	julia.richter@richtersisters.de
	Schuba	Barbara	barbara.schuba@med.uni-muenchen.de
	Speck	Eike	eike.speck@med.uni-muenchen.de
	Stierle	Anna-Lisa	anna@stierle-mail.de
University Hospital of Prague, Prague, Czech Republic	Bruthans	Jan	jan.bruthans@vfn.cz
	Matek	Jan	jan.matek@vfn.cz
	Michálek	Pavel	pavel.michalek@vfn.cz
Radboud University Medical Centre Nijmegen, The Netherlands	Didden	Loes	loes.didden@radboudumc.nl
	Hofland	Jan	jan.hofland@radboudumc.nl
	Kuut	Marieke	marieke.kuut@radboudumc.nl
	Mourisse	Jo	jo.mourisse@radboudumc.nl
Hospital Universitario de la Ribera, Alzira, Spain	Aragon	Sonsoles	aragon_son@gva.es
	Esturi	Rafael	esturi_raf@gva.es
	Miñana	Encarna	minyana_enc@gva.es
	Sanchez	Fernando	sanchez_fergar@gva.es
Department of Anaesthesia, Postoperative ICU, Pain Relief & Palliative Care Clinic, ‘Sotiria’ Chest Diseases Hospital, Athens, Greece	Sfikas	Elaine	elaisfikas@hotmail.com
	Kapezanos	Athanasios	a.kapezanos@yahoo.com
	Papamichail	Konstantinos	kospapam@yahoo.gr
	Toufektzian	Levon	tlevon@gmail.com
	Voyagis	Gregorios	gsvgasman@yahoo.com
Hospital General Universitario of Valencia, Valencia, Spain	Granell Gil	Manuel	mgranellg@hotmail.com
	Vergara Sánchez	Asunción	asuncionvergara@gmail.com
	De Andres	Jose	deandres_jos@gva.es
	Morales Sarabia	Javier	jems.com@gmail.com
	Broseta Lleó	Ana	ana.broseta@gmail.com
	Hernández Laforet	Javier	jaherla@hotmail.com
	Murcia Anaya	Mercedes	merxemurcia@gmail.com
Hospital Álvaro Cunqueiro, Vigo, Spain	Pereira Matalobos	Denis	denispema@gmail.com
	Aguirre Puig	Pilar	pilaraguirrepuig@yahoo.es
Division Anesthesiology and ICU, Department of Thoracic Surgery Jordanovac University Hospital Centre Zagreb, Zagreb,Croatia	Špiček Macan	Jasna	mspicekj@hotmail.com
	Karadza	Vjekoslav	vkaradza@xnet.hr
	Kolaric	Nevenka	nevenkakolaric@yahoo.com
University Medical Centre Ljubljana, Slovenia	Andjelković	Lea	lea.andjelkovic@gmail.com
	Drnovšek Globokar	Mojca	mojca.drnovsek@gmail.com
	Gorjup	Kristina	kristinagorjup@gmail.com
	Mavko	Ana	ana.mavko@gmail.com
	Pirc	Dejan	pirc.dejan@gmail.com
Institutul de Pneumoftiziologie, Bucharest, Romania	Genoveva	Cadar	genovevacadar@hotmail.com
	Istrate	Raluca	raluca_crintea@yahoo.com
	Stoica	Radu	raduati1957@gmail.com
Central Military Emergency University Hospital, Bucharest, Romania	Corneci	Dan	dcorneci@yahoo.com
	Tanase	Narcis Valentin	tanasenv@yahoo.com
Clinic for Anesthesia and Intensive Therapy, Clinical Center Nis, School of Medicine, University of Nis, Nis, Serbia	Radmilo	Jankovic	jankovic.radmilo@gmail.com
	Cvetanovic	Vladan	vladan.cvetanovic@gmail.com
	Dinic	Vesna	vesnadinic1981@gmail.com
	Grbesa	Tijana	grbesatijana@gmail.com
	Jovic	Katarina	katarina.jovic76@gmail.com
	Nikolic	Aleksandar	draleksandarnikolic@hotmail.com
	Stojanovic	Milena	milenastojanoviclaci@gmail.com
	Veselinovic	Ines	inesveselinovic@gmail.com
	Vukovic	Anita	anita83ptr@gmail.com
Merheim Hospital, Cologne, Germany	Wappler	Frank	wapplerf@kliniken-koeln.de
	Defosse	Jerome Michel	defossej@kliniken-koeln.de
	Wehmeier	Stefanie	wehmeiers@kliniken-koeln.de
University Hospital Münster, Department of Anesthesiology, Intensive Care and Pain Medicine, Münster, Germany	Ermert	Thomas	ermert@uni-muenster.de
	Zarbock	Alexander	zarbock@uni-muenster.de
	Wenk	Manuel	manuelwenk@uni-muenster.de
Hospital Marie Lannelongue, Le Plessis-Robinson, France	Ion	Daniela Iolanda	iolandaion@yahoo.com
	Ionescu	Cristian	iraducristi@yahoo.com
Department of Anesthesiology and Intensive Care Medicine, University Hospital Otto von Guericke, Magdeburg, Germany	Schilling	Thomas	thomas.schilling@med.ovgu.de
	Macharadze	Tamar	tamrikomacharadze@hotmail.com
Taichung Veterans General Hospital, Taichung City, Taiwan	Li	Pei-Ching	pei9502@gmail.com
	Chang	Yi-Ting	kikicoco36@gmail.com
Anestesia e Rianimazione, Policlinico Univ. G. Martino, Messina, Italy	Noto	Alberto	dralbert@unime.it
	Calì	Placido	placidocali@alice.it
	Desalvo	Giovanni	giannidesalvo@gmail.com
	Deluca	Raffaele	delucaraffa@gmail.com
	Giofre’	Nicola	ngiofre@hotmail.it

### Additional files

Additional file 1: PROTHOR-Patient information. (DOCX 19 kb)
