# Correction to: Protective ventilation with high versus low positive end-expiratory pressure during one-lung ventilation for thoracic surgery (PROTHOR): study protocol for a randomized controlled trial

**DOI:** 10.1186/s13063-019-3371-y

**Published:** 2019-05-08

**Authors:** T. Kiss, J. Wittenstein, C. Becker, K. Birr, G. Cinnella, E. Cohen, M. R. El Tahan, L. F. Falcão, C. Gregoretti, M. Granell, T. Hachenberg, M. W. Hollmann, R. Jankovic, W. Karzai, J. Krassler, T. Loop, M. J. Licker, N. Marczin, G. H. Mills, M. T. Murrell, V. Neskovic, Z. Nisnevitch-Savarese, P. Pelosi, R. Rossaint, M. J. Schultz, A. Serpa Neto, P. Severgnini, L. Szegedi, T. Vegh, G. Voyagis, J. Zhong, M. Gama de Abreu, M. Senturk, for the PROTHOR investigators and the Research Workgroup PROtective VEntilation Network (PROVEnet) of the European Society of Anaesthesiology (ESA)

**Affiliations:** 1 Department of Anesthesiology and Intensive Care Medicine, Pulmonary Engineering Group, University Hospital Carl Gustav Carus, Technische Universität Dresden, Dresden, Germany; 2 Department of Anesthesia and Intensive Care, OO Riuniti Hospital, University of Foggia, Foggia, Italy; 3 Department of Anesthesiology, The Mount Sinai Hospital, New York, USA; 4 Imam Abdulrahman Bin Faisal University, Dammam, Saudi Arabia; 5 Federal University of São Paulo, Sao Paulo, Brazil; 6 UOC Anestesia e Rianimazione A.O.Universitaria “P. Giaccone”, Dipartimento Di.Chir.On.S, Università degli Studi di Palermo, Palermo, Italy; 7 Hospital General Universitario de Valencia, Valencia, Spain; 8 University Hospital Magdeburg, Magdeburg, Germany; 9 Department of Anesthesiology, Amsterdam UMC, location AMC, Amsterdam, The Netherlands; 10 Clinic for Anesthesia and Intensive Therapy, Clinical Center Nis, School of Medicine, University of Nis, Nis, Serbia; 11 Zentralklinik Bad Berka, Bad Berka, Germany; 12 Thoracic Center Coswig, Coswig, Germany; 13 Department of Anesthesiology and Intensive Care Medicine Clinic, Medical Center, University of Freiburg, Faculty of Medicine, University of Freiburg, Freiburg, Germany; 14 University Hospital Geneva, Geneva, Switzerland; 15 Section of Anaesthetics, Pain Medicine and Intensive Care, Department of Surgery and Cancer, Faculty of Medicine, Imperial College London, London, UK; 16 Department of Anaesthesia, Royal Brompton and Harefield NHS Foundation Trust, Harefield Hospital, Harefield, Middlesex UK; 17 Centre of Anaesthesia and Intensive Care, Semmelweis University, Budapest, Hungary; 18 Department of Anaesthesia and Intensive Care Medicine, Sheffield Teaching Hospitals, Sheffield University, Sheffield, UK; 19 Department of Anesthesiology, Weill Cornell Medicine, New York, USA; 20 Military Medical Academy, Belgrade, Serbia; 21 Penn State Hershey Anesthesiology & Perioperative Medicine, Hershey, USA; 22 Department of Surgical Sciences and Integrated Diagnostics, University of Genoa, Genoa, Italy; 23 IRCCS San Martino Policlinico Hospital, Genoa, Italy; 24 Department of Anaesthesiology, University Hospital Aachen, Aachen, Germany; 25 Department of Intensive Care & Laboratory of Experimental Intensive Care and Anesthesiology (L·E·I·C·A), Academic Medical Center, University of Amsterdam, Amsterdam, The Netherlands; 26 Mahidol-Oxford Tropical Medicine Research Unit (MORU), Mahidol University, Bangkok, Thailand; 27 Department of Critical Care, Hospital Israelita Albert Einstein, São Paulo, Brazil; 28 Dipartimento di Biotecnologie e Scienze della Vita, Università degli Studi dell’Insubria, Varese, Italy; 29 Department of Anesthesiology, Centre Hospitalier Universitaire de Charleroi, Charleroi, Belgium; 30 Department of Anesthesiology and Intensive Care, University of Debrecen, Debrecen, Hungary; 31 Outcomes Research Consortium, Cleveland, USA; 32 Department of Anaesthesia, Postoperative ICU, Pain Relief & Palliative Care Clinic, “Sotiria” Chest Diseases Hospital, Athens, Greece; 33 Department of Anaesthesiology and Critical Care Medicine, University of Patras, Patra, Greece; 34 Department of Anesthesiology, Fudan University Shanghai Cancer Center, Shanghai, China; 35 Department of Oncology, Shanghai Medical College, Fudan University, Shanghai, China; 36 Department of Anaesthesiology and Intensive Care, Istanbul University, Istanbul Medical Faculty, Istanbul, Turkey

**Correction to: Trials**

**https://doi.org/10.1186/s13063-019-3208-8**

After publication of the original article [1], the authors have notified us that two of the collaborator first and last names have been inverted in the “PROTHOR Investigators” appendix table.

The correct information is:

Surname: Spadaro

Name: Savino

Surname Vitali

Name Costanza

The original article has been corrected.
