# Supplementary material for: Protective ventilation with high versus low positive end-expiratory pressure during one-lung ventilation for thoracic surgery (PROTHOR): study protocol for a randomized controlled trial
Source: Trials. 2019 Apr 11;20:213. doi: 10.1186/s13063-019-3208-8 (PMC6460685; doi:10.1186/s13063-019-3208-8)
Supplement: Supplementary file 1 — PROTHOR-Patient information. (DOCX 19 kb) [file 13063_2019_3208_MOESM1_ESM.docx]

**PROTHOR-Patient information**

**PROtective ventilation with high versus low PEEP during one-lung ventilation for THORacic surgery – PROTHOR: A randomized controlled trial**

Dear patient,

you are planned for a surgical procedure with general anesthesia, a procedure which requires mechanical ventilation.

**Background of the study**

During mechanical ventilation, the patient receives air to inhale through positive pressure. During exhalation, this overpressure is reduced and the air can escape from the lungs. As a result of the anesthesia the abdominal muscles are relaxed. Due to this fact, more air escapes from the lungs as in the normal waking state. As a result, lung areas develop that are not filled with air that can impair the function of the lung (so-called "atelectasis"). Patients scheduled for thoracic surgery in particular are at risk of lung function impairment. During anesthesia, this is a challenge for the anesthesist, as well as during the recovery phase after surgery. The anesthetist can improve the lung function and mechanical ventilation parameters by setting a constantly prevailing pressure on the ventilator (so-called "positive end-expiratory pressure", often abbreviated as "PEEP"). This study focuses on the question regarding which level of constant pressure (PEEP) during ventilation under anesthesia should be adjusted in the future. This question has ben insufficiently investigated so far. At present the used PEEP level is rather low. This means that atelectatic lung areas are deliberately accepted as long as the lung function is stable. With a higher PEEP level ventilation can be improved, but the circulation can be impaired (e.b. drop of blood pressure). In this case, specific drugs are necessary to support the patient’s circulatory function. The primary goal of the study is to answer the question whether the setting of a particular PEEP level can prevent pulmonary complications that occur in the recovery phase after the surgery (eg pulmonary infections, oxygen supply). We assume, that a higher PEEP level preserves lung function not only during anesthesia, but also postoperatively.

**Study description**

In a first step, an anesthesist will first determine whether you are suitable for this study by means of a questionnaire. Before the surgical procedure, the study physician will collect informations on your medical history, perform a physical examination and blood sampling. During the operation, you will be ventilated with a high or low PEEP for the entire procedure of surgery. The ventilation method that is chosen for you will be decided by a random lottery, which doctors and patients will have no influence on. Before the end of anesthesia, another blood sample will be taken. On the first day after the operation, a patient interview and a physical examination are performed, which are repeated once a day until the fifth day after the operation and the day of your hospital discharge.

Your discharge from the hospital is not delayed due to participation in the study. No additional examinations after hospital discharge are made.

**Individual benefits, risks and burdens for the patient**

The probability that you will be assigned to the group with the higher respiratory pressure (PEEP) or the group with the lower respiratory pressure (PEEP) is equal, 50% for both cases. A particular advantage for all patients participating in this study is that they can benefit from extended monitoring during and after the operation. Nevertheless, there are different risks and benefits for both groups.

If you are assigned to the low-pressure ventilation group, you will be given the ventilation treatment that is preferred by most of the anesthesists all over the world. During low pressure ventilation, the oxygen content in the blood may be too low so that the respiratory gas mixture or respiratory pressure has to be adapted. A particular advantage of this form of ventilation is that the circulation is somewhat more stable.

During ventilation with higher pressure, the air passages may well be held open, which is likley to help the transfer of oxygen. However, a transient drop in blood pressure may occur, which can be easily treated by the administration of specific drugs. These drugs occasionally lead to a reduction of the heart rate, which does not cause damage. If a situation develops with impaired lung function or depression of the cardiocirculatory system, the applied ventilation pressure will be adapted to restore proper function.

Physical examination does not involve invasive or painful procedures. The study-related blood samples are, as far as possible, linked to the routine blood collection or carried out via existing vascular access. In the case of study-related venipunctions, there is a risk of infections, hematoms and nerve damage in the area of ​​the puncture site or its surroundings. In the blood samples, laboratory tests are carried out, which allow a statement on the function of different organs, including the lungs. Blood samples will be stored in the Department of Anesthesia and Intensive Care (Director: Prof. T. Koch) of the University Hospital Carl Gustav Carus in Dresden and destroyed after 10 years.

**Privacy policy**

The data collected in the study will be saved both electronically and in paper form for at least 10 years in the coordination center for clinical trials in Dresden and partly also in the Department of Anesthesia and Intensive Care of the University Hospital Carl Gustav Carus Dresden (Director: Prof. Dr. T. Koch). This time period can be changed by new laws of the Federal Republic of Germany. Data access is granted to the staff of the KKS Dresden and the investigators of the study in Dresden, whereby all parties must comply with the german data safety and privacy regulations. For this case your consent is required. The data will be processed by special software and statistically evaluated. In data processing, the identity of the patients is encrypted by means of a number code (pseudonymization). This code can be passed on to the relevant monitoring authorities. For inspection purposes the access to the encrypted data can be granted, but the person entitled to access is obliged to maintain secrecy.It is planned to publish the study results in one or more scientific journals. In this case, due to the pseudonymization conclusions on individual patients will not be possible.You have the right to access and to correct your data collected in the study. Informing your family practitioner about participation in the study is desirable, but requires your explicit consent.

**Voluntariness and consent withdrawal**

Participation in the study is on a voluntary basis and the rejection does not bring any disadvantages for further treatment of the patient. Data which may have been collected up to the date of consent withrawal shall not be passed on, even in an anonymous form, and will not be included in the evaluation. The consent you have given may be revoked at any time without giving reasons!
